# Admixture mapping analysis in the context of GWAS with GAW18 data

**DOI:** 10.1186/1753-6561-8-S1-S3

**Published:** 2014-06-17

**Authors:** Mengjie Chen, Can Yang, Cong Li, Lin Hou, Xiaowei Chen, Hongyu Zhao

**Affiliations:** 1 Program in Computational Biology and Bioinformatics, Yale University, New Haven, CT 06520, USA; 2 Department of Biostatistics, Yale School of Public Health, Yale University, New Haven, CT 06520, USA

## Abstract

Admixture mapping is a disease-mapping strategy to identify disease susceptibility variants in an admixed population that is a result of mating between 2 historically separated populations differing in allele frequencies and disease prevalence. With the increasing availability of high-density genotyping data generated in genome-wide association studies, it is of interest to investigate how to apply admixture mapping in the context of the genome-wide association studies and how to adjust for admixture in association tests. In this study, we first evaluated 3 different local ancestry inference methods, LAMP, LAMP-LD, and MULTIMIX. Then we applied admixture mapping analysis based on estimated local ancestry. Finally, we performed association tests with adjustment for local ancestry.

## Background

In human genetics studies, several approaches are commonly used to identify disease risk variants, including linkage analysis, association analysis, and admixture mapping. Among these 3 methods, admixture mapping can fill a niche between family based linkage analysis and population-based association analysis, when the disease-causing variants differ in frequency between different ethnic groups because of drift or selection. This approach has been successfully applied in recent studies of African Americans [1] and Mexican Americans [2], implicating susceptibility regions for prostate cancer and type 2 diabetes that are associated with ancestry. Of relevance for Genetic Analysis Workshop 18 (GAW18), epidemiological studies have found that rates of hypertension vary markedly in different regions and ethnic groups, suggesting that admixture mapping may be a viable approach to analyzing the GAW18 Mexican American cohort. In this study, we first compared 3 local ancestry inference methods based on several metrics. With the estimated local ancestry, we then performed admixture mapping. Finally, we performed association tests with adjustment for local ancestry.

## Methods

### Data set, reference panels, and preprocessing

All analyses presented in this paper are based on the genotyping data of 109 unrelated individuals with blood pressure information from GAW18. Two individuals, T2DG1101320 and T2DG0800490, were excluded because of their high genotype missing rates. For association analysis with quantitative traits, we used log-transformed systolic and diastolic blood pressure measurements at the second examination of each individual. For binary traits, that is, case or control, all individuals diagnosed with hypertension at least once were classified as cases, whereas others were classified as controls. Because of the methodological purpose of our analysis, we performed the analyses only on chromosome 3.

We utilized CEU and YRI samples of release 27 of merged phases II and III of the International Haplotype Map Project (HapMap) for European and African ancestry, respectively, and the Human Genome Diversity Project (HGDP) samples from the Americas for Native American ancestry, which include 6 Colombian, 13 Karitiana, 22 Maya, 14 Pima, and 8 Surui individuals (denoted as NA). We extracted markers that were present in both the reference panels and the GAW18 subjects and then removed markers with missingness greater than 20%, resulting in a set of 37,438 single-nucleotide polymorphisms (SNPs). Inconsistency in the strand orientation was observed among data from HapMap, HGDP, and GAW18. We realigned HGDP and GAW18 to the orientation of HapMap, resulting in 7004 of the 37,438 SNPs being recoded.

### Global and local ancestry estimation

We performed supervised global ancestry estimation of chromosome 3 using ADMIXTURE [3], with the number of ancestral populations fixed at 3. Global estimates were used as a reference metric to assess the performance of local ancestry estimates. The performance of ADMIXTURE depends highly on the number of markers used. Generally, more markers are needed to perform adequate genome-wide association studies (GWAS) correction than to depict population structure. As a rule of thumb, 100,000 markers (genome-wide) are necessary to perform GWAS correction when populations are within a continent (the context of GAW18). To fully harness the ancestral information from markers, we used 37,348 SNPs for chromosome 3. Thus, we think the global ancestry estimation from ADMIXTURE is of high quality.

We used LAMP, LAMP-LD [4], and MULTIMIX [5] to estimate local ancestry. To apply LAMP, we first constructed an ancestry-informative marker (AIM) panel based on the F-statistic (F_st_), a commonly used measure of genetic diversity across populations. To calculate F_st_, we used allele frequencies for CEU and YRI from HapMap release 27, as well as allele frequencies for Mayan (MAY) and Pima (PMA) from the Allele FREquency Database (ALFRED) [6]. Sets of AIMs were selected so that for each SNP, (a) allele frequency was similar in Mayan and Pima Indians (Fst_MAY−PMA _<0.1); (b) allele frequency was different in CEU and YRI (F_stCEU−MAY _>0.2 and F_stCEU−PMA _>0.2 and F_stYRI−MAY _>0.2 and F_stYRI−PMA _>0.2); and (c) LD *r*^2 ^<0.1 for each pair of selected SNPs (this step was automatically performed in LAMP). This resulted in 522 AIMs in total. We ran LAMP in the LAMPANC mode inputting allele frequencies of CEU, YRI, and NA, respectively, with the following configuration parameters: mixture proportions (alpha) = 0.6, 0.1, 0.3; number of generations since admixture (g) = 10; recombination rate (r) = 1e-8; fraction of overlap between adjacent windows (offset) = 0.2; and *r*^2 ^threshold (ldcutoff) = 0.1.

To apply LAMP-LD, we first phased the reference panel using the SHAPEIT software [7] with default settings. We then ran LAMP-LD on the GAW18 samples. Considering that LAMP-LD used window-based processing in its model, we tested the effects of different window sizes (150, 100, 75, and 50). We applied MULTIMIX to both phased (by SHAPEIT) and unphased GAW18 samples with phased reference. For phased samples, we used the MULTIMIX_EM algorithm with resolving step. For unphased samples, we used the MULTIMIX_MCMCgeno algorithm with misfitting probabilities equal to the estimation from MULTIMIX_EM.

### Estimation of the number of generations since admixture

Given the number of ancestry segments (*A*) of each individual, we estimate the number of generations (*N*) since admixture at *N = A/4a(1-a)L*, where *a *is the admixture proportion of European ancestry, *4a(1-a) *is the number of expected recombination events in a diploid individual, and *L *is the length of genetic map in morgans (2.217 morgans on chromosome 3).

### Admixture mapping analysis

We used a linear regression model similar to the model proposed by Zhu *et al*. [8] for admixture analysis. Specifically, let $y_{i}$ be the residual trait value of individual i after adjusting for age and gender. Let $S_{ij}$ be the European/Native American ancestry at the *j*th marker, and ${\bar{S}}_{i}$ be the average of the European/Native American ancestry of individual i. We tested the null hypothesis $\beta_{2}=0$ on the model $y_{i}=\beta_{0}+\beta_{1}{\bar{S}}_{i}+\beta_{2}\left(S_{ij}-{\bar{S}}_{i}\right)+\varepsilon_{i}$. We selected SNPs whose false discovery rate (FDR) adjusted *p*-value was less than 0.05.

### Association analysis with adjustment for local ancestry

We propose an association test with adjustment for local ancestry based on the model $y_{i}=\beta G_{ij}+\sum_{k=1}^{K}\beta_{k}\alpha_{ijk}q_{k}+\varepsilon_{i}$, where $y_{i}$ is the residual trait value of individual i as defined above, $G_{ij}$ is half of the number of nonreference alleles, $\alpha_{ijk}$ is the local ancestral proportion for the *k*th ancestral population of individual i at the *j*th marker, and $q_{k}$ is the allele frequency of the nonreference allele for the *k*th ancestral population. We tested for association with the null hypothesis:β=0. We selected SNPs whose FDR-adjusted *p*-value was less than 0.05.

## Results and discussion

### Comparisons of local ancestry inference methods

Among the 3 methods we compared, LAMP represents traditional methods that infer local ancestry on a predefined AIM panel with several thousand SNPs across the entire genome. It does not use linkage disequilibrium (LD) information and only works when the SNP density is not high. LAMP-LD and MULTIMIX represent methods taking into account background LD and are capable of multiway admixture deconvolution. Phased reference panels are required for LAMP-LD. Compared with LAMP-LD, MULTIMIX is more flexible with input data. It can handle both phased and unphased sample genotypes, as well as phased and unphased reference panels. Both methods involve a window-based processing procedure; MULTIMIX requires an additional boundary-resolving step with a larger computational burden, whereas LAMP-LD resolves the boundaries internally with better efficiency.

**Figure 1:**
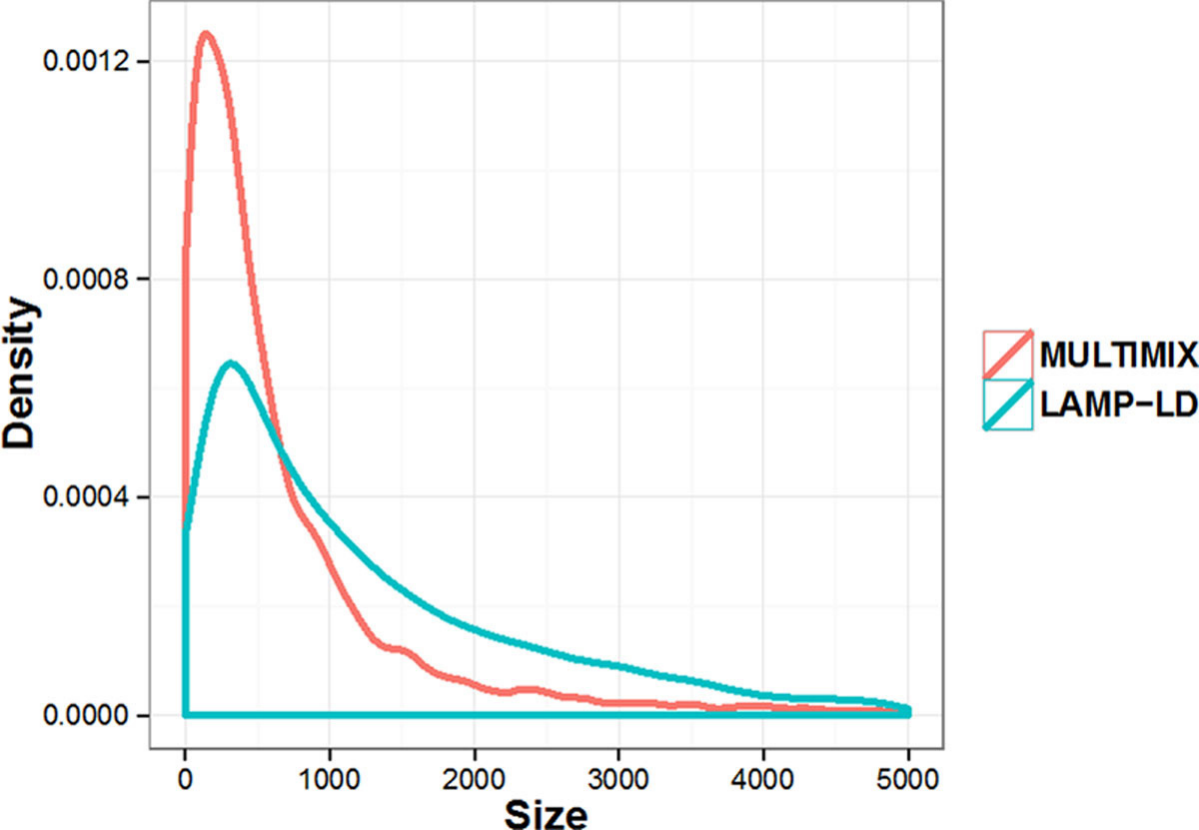
**Density plot for sizes of ancestry segments from LAMP-LD and MULTIMIX**. Only segments with sizes under 5000 SNPs are shown.

Table 1 summarizes the results from these 3 methods. LAMP-LD achieved the highest correlation (0.989) with the global estimates from ADMIXTURE. LAMP-LD and MULTIMIX, based on unphased genotype data, led to similar estimates on the number of ancestry segments, which suggests that the number of generations since admixture is between 10 and 12. This is consistent with previous reports that Latino and Hispanic populations have been admixed within the past 10 generations. We note that the number of ancestry segments from MULTIMIX using phased data is double that from the unphased data. This increment results from more inferred segments with smaller sizes from the phased haplotypes (Figure 1). Therefore, additional simulation studies are needed to investigate the robustness of the inference results given the uncertainty in haplotype phasing.

**Table 1:** Comparisons of different local ancestry inference methods.

Method^1^	Input genotype	SNP density	Computing time (minutes)^2^	Number of ancestry segments		Correlation with admixture
					
				Mean	SD	
LAMP	Unphased	Low	<1	6.00	1.78	0.920
LAMP-LD	Unphased	High	128	25.49	7.27	0.989
MULTIMIX	Unphased	High	20	22.40	17.5	0.923
MULTIMIX	Phased	High	20 + 560^3^	49.13	18.87	0.975

^1^Only results from runs with window size of 100 are listed in Table 1.

^2^One hundred seven samples were tested on a PowerEdge M600 node 2.33 GHz with 16 GB RAM.

^3^Additional 560 minutes were used to resolve the boundaries.

### Comparisons of local ancestry estimates

We further compared local ancestry estimates from LAMP-LD and MULTIMIX with phased data (Table 2). Using global estimates from ADMIXTURE as reference, we first assessed the reliability of local ancestry estimates at a global level. LAMP-LD produced very stable estimates across different window sizes, with the mean Pearson correlation around 0.989 and 4% of SNP ancestry "mislabelled." MULTIMIX achieved better accuracy with smaller window sizes. When the window size decreased from 150 to 50 SNPs, the mislabelled rate dropped from 8% to 4.5%. Using the same window size, LAMP-LD always outperformed MULTIMIX.

**Table 2:** Comparisons of local estimates from LAMP-LD and MULTIMIX.

Method	Window size (SNPs)	Mean correlation^1^	SD of correlation^1^	Mean deviation (%)^1^	SD of deviation (%)^1^	Diploid inconsistency (%)
LAMP-LD	150	0.988	0.043	4.0	3.6	19.8
MULTIMIX	150	0.959	0.105	8.0	5.8	

LAMP-LD	100	0.989	0.041	3.9	3.4	18
MULTIMIX	100	0.975	0.100	6.0	5.2	

LAMP-LD	75	0.989	0.041	3.9	4.1	16.6
MULTIMIX	75	0.974	0.108	6.0	4.5	

LAMP-LD	50	0.989	0.043	3.7	3.2	14.2
MULTIMIX	50	0.985	0.072	4.5	3.5	

^1^Comparisons with global estimates from ADMIXTURE. Deviation is defined as the percentage of inconsistent SNPs.

**Figure 2:**
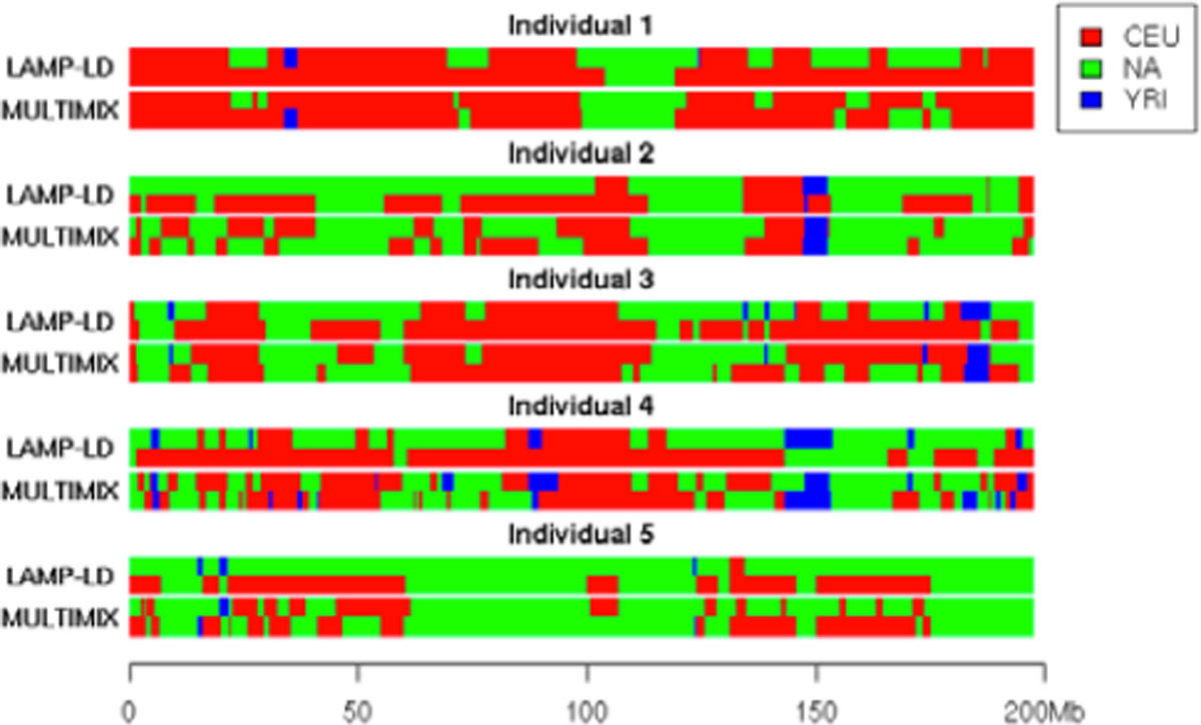
**Local ancestry estimates of 5 individuals on chromosome 3**.

Although estimates from MULTIMIX and LAMP-LD are comparable at the global level, there was appreciable difference at the local level (Figure 2). We further checked differences on the estimates of 6 diploid types (CEU-YRI, YRI-NA, CEU-NA, CEU-CEU, YRI-YRI, NA-NA) (see Table 2). For example, when the window size was fixed to 100 SNPs, 18% of the SNPs had inconsistent diploid inferences between the 2 methods, which led to differences in downstream admixture mapping and association analysis results.

### Admixture mapping analysis and association test

We used local ancestry estimates from MULTIMIX and LAMP-LD with window size 100 for both admixture mapping analyses and association tests. No SNP's adjusted FDR *p*-value is lower than 0.05 for all the tests. This may be as a result of the low power because of the small sample size and/or the lack of genomic regions affecting these traits.

Overall, the admixture mapping analysis for local ancestry is underpowered. The genomic control factors for the test on European ancestry using LAMP and MULTIMIX amounted to 0.894 and 0.936, respectively. We further compared the test statistics (*t*) from admixture mapping analyses using different local ancestry estimates. In the association tests of diastolic blood pressure with European ancestry, we found 694 SNPs with a *t*-statistic higher than 3 based on MULTIMIX estimates, compared to 68 for LAMP-LD estimates. We used permutation to assess the significance of this association. Specifically, we permuted the traits and subsequently fitted the regression model 3000 times. For each regression we calculated the number of SNPs with a *t*-statistic greater than 3. By comparing the observed *t*-value with the distribution of the statistic from the permutations, we obtained a *p*-value of 0.027 for MULTIMIX estimates and a *p*-value of 0.2 for LAMP-LD estimates. This indicates the inconsistent inferences between MULTIMIX and LAMP-LD may lead to different conclusions in downstream analyses.

The small number of investigated individuals was a limitation of the present study. For example, assuming a 9.75% exposure probability among controls (minor allele frequency 5% and dominant penetrance model), a type I error equal to 0.05/37,438, and a sample size of 36 cases and 72 controls (the hypertension prevalence in the sample was 33%), the present study had 80% power to identify a genotype relative risk of 14.4. The low statistical power clearly limits the detection of effects attributable to ancestry adjustment.

## Conclusions

Although many methods have been proposed to infer local ancestry in the last several years, most of them are not applicable to 3-way admixed Latino and Hispanic populations, nor can they account for background LD. LAMP-LD and MULTIMIX are newly developed methods to address these challenges. This report shows that both methods performed better than LAMP in the context of GWAS with densely spaced markers. Using global estimates from ADMIXTURE as a standard, this report shows that both methods achieved high accuracy of ancestry estimation at the global level (greater than 95%). However, 18% of the SNPs had different ancestry inferences between the 2 methods. MULTIMIX with phased samples produces much smaller ancestry segments, which is a major cause of the discrepancy at the local level. The statistical properties of MULTIMIX need to be further studied. Consequently, multiway admixture deconvolution at the local level is still a challenging problem.

It has been shown that local ancestry at a SNP might confound with the association signal. Ignoring this could lead to spurious associations [9]. Although no significant association was detected in the present exercise, we note that local ancestry is an important facet of population stratification, and integrating the local heterogeneity into association tests is necessary for admixed samples.

## Competing interests

The authors declare that they have no competing interests.

## Authors' contributions

MC and HZ designed the overall study; MC, CY, CL, XC, and LH conducted statistical analyses; and MC and HZ drafted the manuscript. All authors read and approved the final manuscript.

## References

[B1] Reich D Patterson N De Jager PL McDonald GJ Waliszewska A Tandon A Lincoln RR DeLoa C Fruhan SA Cabre P A whole-genome admixture scan finds a candidate locus for multiple sclerosis susceptibility Nat Genet 2005 37 1113 1118 10.1038/ng1646 16186815

[B2] Adler S Pahl M Abboud H Nicholas S Ipp E Seldin M Mexican-American admixture mapping analyses for diabetic nephropathy in type 2 diabetes mellitus Semin Nephrol 2010 30 141 149 10.1016/j.semnephrol.2010.01.005 20347643 PMC2967569

[B3] Alexander DH Novembre J Lange K Fast model-based estimation of ancestry in unrelated individuals Genome Res 2009 19 1655 1664 10.1101/gr.094052.109 19648217 PMC2752134

[B4] Baran Y Pasaniuc B Sankararaman S Torgerson DG Gignoux C Eng C Rodriguez-Cintron W Chapela R Ford JG Avila PC Fast and accurate inference of local ancestry in Latino populations Bioinformatics 2012 28 1359 1367 10.1093/bioinformatics/bts144 22495753 PMC3348558

[B5] Churchhouse C Marchini J Multi-way admixture deconvolution using phased or unphased ancestral panels Genet Epidemiol 2013 37 1 12 10.1002/gepi.21692 23136122

[B6] The ALlele FREquency Database http://alfred.med.yale.edu/alfred/AboutALFRED.asp

[B7] Delaneau O Marchini J Zagury JF A linear complexity phasing method for thousands of genomes Nat Methods 2011 9 179 181 10.1038/nmeth.1785 22138821

[B8] Zhu X Young JH Fox E Keating BJ Franceschini N Kang S Tayo B Adeyemo A Sun YV Li Y Combined admixture mapping and association analysis identifies a novel blood pressure genetic locus on 5p13: contributions from the CARe consortium Hum Mol Genet 2011 20 2285 2295 10.1093/hmg/ddr113 21422096 PMC3090198

[B9] Wang X Zhu X Qin H Cooper RS Ewens WJ Li C Li M Adjustment for local ancestry in genetic association analysis of admixed populations Bioinformatics 2011 27 5 670 677 10.1093/bioinformatics/btq709 21169375 PMC3042179

